# Supplementary material for: Leisure engagement in older age is related to objective and subjective experiences of aging
Source: Nat Commun. 2024 Feb 19;15:1499. doi: 10.1038/s41467-024-45877-w (PMC10876530; doi:10.1038/s41467-024-45877-w)
Supplement: Supplementary file 1 — Supplementary Information [file 41467_2024_45877_MOESM1_ESM.pdf]

Supplementary Materials

Cross-sectional associations

As in the longitudinal analyses, we used an outcome-wide approach [1,2] to test the associations between frequency of engagement in each leisure domain (physical, creative, cognitive, and community activities) and aging experiences in regression models. Type of regression was determined by the outcome; negative binomial regression was used for count outcomes (to deal with overdispersion), linear regression for continuous, logistic regression for binary, and ordered logistic regression for ordinal outcomes. Cross-sectional associations between leisure engagement and each experience of aging at baseline were adjusted for all demographic, socioeconomic, and neighborhood covariates. Here we describe the results from adjusted analyses (Figure 1; Table S5), but unadjusted analyses are also included below (Table S4).

Physical activities

Concurrently, more frequent engagement in physical activities (e.g., sport/exercise, walking) was positively associated with all aspects of aging except grip strength and systolic blood pressure (Figure S1; Table S5). Older adults who did physical activities more frequently had better daily functioning and physical fitness, fewer long-term physical health problems, better heart health, lower weight, better sleep, and more positive perceptions of health. For example, more frequent engagement was associated with fewer difficulties with mobility (odds ratio [OR]=0.73, 95% confidence interval [CI]=0.71-0.76) and activities of daily living (ADLs; incidence rate ratio [IRR]=0.79, 95% CI=0.75-0.84), lower odds of being overweight or living with obesity (BMI; OR=0.80, 95% CI=0.77-0.83), better static balance (OR=1.14, 95% CI=1.09-1.19), and a smaller waist circumference (β=-0.15, 95% CI=-0.16 to -0.13).

Creative activities

More frequent engagement in creative activities (e.g., gardening, needlework, hobbies) was concurrently associated with better outcomes in most aspects of daily functioning, physical fitness, long-term physical health problems, sleep, and subjective perceptions of health. The notable exception was heart health, in which more engagement was only associated with higher odds of high systolic and diastolic blood pressure. Aside from this, more frequent creative engagement was most strongly associated with fewer difficulties with instrumental activities of daily living (IADLs; IRR=0.69, 95% CI=0.62-0.77), ADLs (IRR=0.81, 95% CI=0.75-0.89), and mobility (OR=0.88, 95% CI=0.84-0.92), higher odds of good static balance (OR=1.14, 95% CI=1.07-1.21), fewer perceived difficulties with balance (OR=0.89, 95% CI=0.85-0.93), and lower odds of not feeling rested after sleep (OR=0.89, 95% CI=0.85-0.94).

Cognitive activities

Concurrently, engaging in cognitive activities (e.g., reading, writing, games) was only associated with positive outcomes for subjective perceptions of health, namely lower odds of rating eyesight as poor (OR=0.93, 95% CI=0.88-0.97). However, doing cognitive activities more frequently was also associated with higher odds of high diastolic blood pressure (OR=1.08, 95% CI=1.02-1.14) and being overweight or living with obesity according to BMI (OR=1.15, 95% CI=1.09-1.21), as well as a larger waist circumference (β=0.09, 95% CI=0.06-0.12).

Community activities

More frequent engagement in community activities (e.g., volunteering, educational courses, sports/social clubs) was concurrently associated with better outcomes in some aspects of heart health, sleep, and subjective perceptions of health. Specifically, community activities were associated with lower odds of high systolic blood pressure (OR=0.92, 95% CI=0.86-0.98), not feeling rested after sleep (OR=0.93, 95% CI=0.87-1.00), rating eyesight as poor (OR=0.93, 95% CI=0.88-1.00), and perceived difficulty with balance (OR=0.93, 95% CI=0.88-0.99).

Sensitivity analyses

Adjustment for baseline health and health behaviors

In this sensitivity analysis, we adjusted for additional health-related confounders that are likely related to both leisure engagement and experiences of aging but are also likely to lie on the causal pathway between leisure engagement and aging outcomes. Including these confounders in the main analyses could therefore block a potential pathway between leisure activities and subsequent health. Health factors were cognition (a summary score on immediate and delayed word recall tasks and counting, naming, and vocabulary mental status indices; 0-35), depressive symptoms (measured on the 8-item Center for Epidemiologic Studies Depression Scale; 0-8), number of conditions taking prescription medication for (from cholesterol, joint or muscle pain, allergies asthma or breathing, stomach problems, sleep, anxiety or depression; 0-6), diagnosis of emotional, nervous, or psychiatric problems (yes, no), and self-rated general health (excellent, very good, good, fair, poor). These factors were measured at baseline due to the high proportion of missing data before baseline. Health behaviors, measured two years before baseline, were number of days per week participant drinks alcohol (0-7) and whether participant currently smokes (yes, no).

Physical activities
More frequent engagement in physical activities was associated with some aspects of daily functioning, physical fitness, long-term physical health problems, and subjective perceptions of health eight years later (Table S6). The strongest associations were for better static balance (odds ratio [OR]=1.08, 95% confidence interval [CI]=1.04-1.12), fewer perceived difficulties with balance (OR=0.95, 95% CI=0.92-0.98), fewer difficulties with mobility (OR=0.91, 95% CI=0.88-0.94) and activities of daily living (ADLs; incidence rate ratio [IRR]=0.95, 95% CI=0.91-1.00), and lower odds of chronic health conditions (OR=0.93, 95% CI=0.90-0.96). There was no evidence for associations with heart health, weight, or sleep.

Creative activities
More frequent engagement in creative activities was associated with some aspects of daily functioning, physical fitness, and sleep eight years later. The strongest associations were fewer difficulties with ADLs (IRR=0.91, 95% CI=0.85-0.96) and instrumental activities of daily living (IADLs; OR=0.92, 95% CI=0.86-0.98), higher odds of good static balance (OR=1.07, 95% CI=1.01-1.13), and lower odds of using sleep medication (OR=0.88, 95% CI=0.81-0.95). However, there was no longitudinal evidence that creative engagement was associated with subsequent long-term physical health problems, heart health, weight, or subjective perceptions of health.

Cognitive activities
There was only evidence for longitudinal associations between engaging in cognitive activities more frequently and lower odds of not feeling rested after sleep (OR=0.93, 95% CI=0.89-0.98) and rating eyesight as poor (OR=0.94, 95% CI=0.89-0.98).

Community activities
There was no evidence for protective associations between more frequent engagement in community activities and aging experiences eight years later. However, more frequent community engagement was associated with higher odds of chronic health conditions eight years later (OR=1.12, 1.05-1.20).

Baseline measure of outcome omitted

In this sensitivity analysis, we repeated the main longitudinal analyses, adjusted for demographic, socioeconomic, and neighborhood covariates. To address concerns about potential biases arising from controlling for the outcome at baseline, we assessed the associations between leisure engagement and experiences of aging eight years later without

adjusting for experiences of aging at baseline (Table S7). Evidence for associations between leisure activities and experiences of aging remained similar, albeit slightly stronger for physical, creative, and cognitive activities (but not community activities).

More frequent engagement in physical activities was beneficially associated with all outcomes except systolic and diastolic blood pressure, for which there was no evidence of an association. More frequent engagement in creative activities was beneficially associated with all aspects of daily functioning, sleep, and subjective perceptions of health, and all aspects of physical fitness except falls, for which there was no evidence. Within long-term physical health problems, it was associated with fewer chronic health conditions. However, it was also associated with higher odds of having high diastolic blood pressure. More frequent engagement in cognitive activities was associated with better lung function and lower odds of not feeling rested after sleep, rating eyesight as poor, and rating hearing as poor. However, cognitive activities were also associated with higher odds of being overweight or living with obesity (according to BMI) and a larger waist circumference. Finally, there was only evidence that more frequent engagement in community activities was associated with lower odds of rating eyesight as poor.

Assessing different levels of leisure engagement

In this sensitivity analysis, we repeated the main longitudinal analyses, adjusted for demographic, socioeconomic, and neighborhood covariates. To provide a more comprehensive picture, we assessed the associations between several levels of leisure engagement and experiences of aging eight years later (Tables S8A and S8B). To do this, we categorized the domains of leisure activities into engagement frequencies, namely no, monthly, and weekly engagement. No engagement was the reference category for all exposures. This indicated that, for physical activities, most of the associations with experiences of aging were driven by more frequent weekly engagement. There were fewer associations for participants who only engaged monthly. In contrast, for creative activities, findings were more similar across monthly and weekly engagement frequencies, showing less of a dose-response relationship. This suggests that any level of engagement in creative activities could potentially be beneficial.

Participants with major chronic conditions at baseline excluded

Finally, due to concerns around reverse causation, we also limited the sample to participants without chronic health conditions at baseline (Table S9). This resulted in small sample sizes (n=395 to n=1460), meaning there was very little evidence for associations between leisure engagement and experiences of aging. Engagement in physical activities was only associated with lower odds of difficulties with mobility, chronic health conditions, and rating hearing as poor. Creative activities were only associated with lower odds of difficulties with ADLs and using sleep medication, as well as better strength and motor function. Neither cognitive nor community activities were associated with any experiences of aging. However, for all domains of leisure engagement, coefficients and confidence intervals were generally in line with the main analyses (Table S3).

Supplementary Tables

Table S1. Sample in which each outcome was measured, and type of regression model used for analyses.

Outcome	HRS sample	N	Model
**Daily functioning**			
Difficulties with ADLs	Core survey	8771	Negative binomial
Difficulties with IADLs	Core survey	8771	Negative binomial
Difficulties with mobility	Core survey	8771	Ordered logistic
**Physical fitness**			
Strength	Core survey	8771	Negative binomial
Gross motor function	Core survey	8771	Negative binomial
Fine motor function	Core survey	8771	Negative binomial
Falls	Aged 65+	4643	Logistic
Gait speed	Physical measures and aged 65+	4131	Linear
Lung function	Physical measures	7940	Linear
Grip strength	Physical measures	7940	Linear
Static balance	Physical measures	7940	Ordered logistic
**Long-term physical health problems**			
Chronic health conditions	Core survey	8771	Ordered logistic
Degree of persistent pain	Core survey	8771	Ordered logistic
**Heart health**			
Systolic blood pressure	Physical measures	7940	Ordered logistic
Diastolic blood pressure	Physical measures	7940	Ordered logistic
Pulse	Physical measures	7940	Linear
**Weight**			
BMI	Physical measures	7940	Ordered logistic
Waist circumference	Physical measures	7940	Linear
**Sleep**			
Uses sleep medication	Core survey	8771	Logistic
How often does not feel rested	Core survey	8771	Ordered logistic
**Subjective perceptions of health**			
Poor eyesight	Core survey	8771	Ordered logistic
Poor hearing	Core survey	8771	Ordered logistic
Perceived difficulty with balance	Core survey	8771	Ordered logistic

Table S2. Proportion of missing data on covariates and outcomes before imputation.

**Variable**	**Total sample size**	**Complete data**	
		Baseline n (%)	Follow-up n (%)
Difficulties with ADLs	8771	0	7 (<1%)
Difficulties with IADLs	8771	0	8 (<1%)
Difficulties with mobility	8771	0	0
Strength	8771	0	0
Gross motor function	8771	0	0
Fine motor function	8771	0	3 (<1%)
Falls	4643	10 (<1%)	11 (<1%)
Gait speed	4131	549 (13%)	648 (16%)
Lung function	7940	689 (9%)	603 (8%)
Grip strength	7940	739 (9%)	751 (9%)
Static balance	7940	720 (9%)	814 (10%)
Chronic health conditions	8771	0	0
Degree of persistent pain	8771	4 (<1%)	19 (<1%)
Systolic blood pressure	7940	827 (10%)	655 (8%)
Diastolic blood pressure	7940	827 (10%)	655 (8%)
Pulse	7940	827 (10%)	655 (8%)
BMI	7940	915 (12%)	942 (12%)
Waist circumference	7940	697 (9%)	717 (9%)
Uses sleep medication	8771	3 (<1%)	27 (<1%)
How often does not feel rested	8771	6 (<1%)	20 (<1%)
Poor eyesight	8771	9 (<1%)	23 (<1%)
Poor hearing	8771	2 (<1%)	10 (<1%)
Perceived difficulty with balance	8771	7 (<1%)	27 (<1%)
Age	8771	0	-
Gender	8771	0	-
Marital status	8771	0	-
Race/ethnicity	8771	2 (<1%)	-
Educational attainment	8771	31 (<1%)	-
Employment status	8771	0	-
Pension status	8771	0	-
Total household income	8771	0	-
Total assets	8771	0	-
Household size	8771	0	-
Neighborhood safety	8771	40 (<1%)	-
Neighborhood physical disorder	8771	133 (2%)	-
Neighborhood social cohesion	8771	139 (2%)	-

Table S3. Sample characteristics at baseline.

**Covariate**	**Proportion**
Female	55%
Married or cohabiting	71%
Race/ethnicity	
White/Caucasian	85%
Black/African American	10%
Other	5%
Educational attainment	
Less than high school	12%
High school	52%
College	23%
Postgraduate	13%
Employment status	
Employed	42%
Retired	48%
Not working	10%
Has a pension	80%
Neighborhood safety excellent/good	92%
	**Mean (SE)**
Age	63.18 (0.10)
Total household income (USD)	81214.79 (1485.41)
Total assets	547151.80 (14694.27)
Household size	0.62 (0.02)
Neighborhood physical disorder	2.42 (0.02)
Neighborhood social cohesion	5.51 (0.02)

Note. Characteristics presented for full sample (n=8771). Results weighted and based on 20 imputed datasets.

Table S4. Descriptive statistics for outcomes at baseline and follow-up.

Outcome	N	Baseline	Follow-up
		Mean (SE) or proportion	
Difficulties with ADLs	8771	0.19 (0.01)	0.33 (0.01)
Difficulties with IADLs	8771	0.15 (0.01)	0.29 (0.01)
Difficulties with mobility	8771		
0		42%	30%
1		34%	34%
2		12%	15%
3		7%	11%
4		5%	10%
Strength	8771	2.22 (0.01)	0.23 (0.01)
Gross motor function	8771	3.80 (0.01)	3.63 (0.01)
Fine motor function	8771	2.87 (0.01)	2.80 (0.01)
Has had a fall	4643	34%	41%
Gait speed	4131	33.41 (0.03)	43.33 (0.05)
Lung function	7940	383.02 (1.95)	342.33 (2.03)
Grip strength	7940	31.18 (0.16)	26.67 (0.15)
Static balance	7940		
None		1%	2%
Side-by-side only		4%	10%
Semi-tandem		19%	28%
Tandem		76%	61%
Chronic health conditions	8771		
0		20%	9%
1		28%	19%
2		26%	25%
3		16%	23%
4		7%	14%
5		3%	10%
Degree of persistent pain	8771		
None		65%	60%
Mild		12%	12%
Moderate		18%	22%
Severe		5%	6%
Systolic blood pressure	7940		
Normal		35%	35%
Elevated		23%	23%
Hypertension stage 1		19%	18%
Hypertension stage 2		23%	23%
Diastolic blood pressure	7940		
Normal/elevated		54%	69%
Hypertension stage 1		30%	23%
Hypertension stage 2		17%	9%
Pulse	7940	69.52 (0.17)	68.57 (0.16)
BMI	7940		
Underweight/healthy weight		20%	21%
Overweight		36%	36%
Obesity		45%	43%
Waist circumference	7940	39.89 (0.09)	40.54 (0.09)
Uses sleep medication	8771	21%	13%
How often does not feel rested	8771		
Most of the time		56%	55%
Sometimes		30%	30%
Rarely/never		14%	14%

Eyesight	8771		
Excellent		12%	9%
Very good		31%	28%
Good		42%	42%
Fair		13%	15%
Poor		3%	5%
Blind		<1%	<1%
Hearing	8771		
Excellent		20%	14%
Very good		30%	27%
Good		33%	37%
Fair		13%	17%
Poor		4%	5%
Perceived difficulty with balance	8771		
Never		44%	28%
Rarely		32%	36%
Sometimes		18%	25%
Often		6%	11%

Note. Results weighted and based on 20 imputed datasets.

Table S5. Unadjusted regression models testing longitudinal associations between leisure engagement and experiences of aging eight years later.

		Physical activities		Creative activities		Cognitive activities		Community activities	
	Coef	Coef (95% CI)	p	Coef (95% CI)	p	Coef (95% CI)	p	Coef (95% CI)	p
**Daily functioning**									
Difficulties with ADLs	IRR	**0.82 (0.79, 0.85)**	**<0.001**	**0.83 (0.78, 0.88)**	**<0.001**	0.97 (0.91, 1.03)	0.333	0.96 (0.88, 1.05)	0.421
Difficulties with IADLs	IRR	**0.87 (0.84, 0.91)**	**<0.001**	**0.84 (0.78, 0.89)**	**<0.001**	0.97 (0.92, 1.03)	0.389	0.95 (0.86, 1.04)	0.247
Difficulties with mobility	OR	**0.75 (0.72, 0.77)**	**<0.001**	**0.93 (0.89, 0.97)**	**0.001**	**1.09 (1.04, 1.14)**	**<0.001**	**0.90 (0.85, 0.96)**	**0.001**
**Physical fitness**									
Strength	IRR	**1.07 (1.06, 1.07)**	**<0.001**	**1.02 (1.01, 1.04)**	**<0.001**	**0.99 (0.97, 1.00)**	**0.032**	**1.03 (1.01, 1.05)**	**<0.001**
Gross motor function	IRR	**1.02 (1.02, 1.03)**	**<0.001**	**1.02 (1.01, 1.02)**	**<0.001**	1.00 (0.99, 1.00)	0.744	**1.01 (1.00, 1.02)**	**0.001**
Fine motor function	IRR	**1.01 (1.01, 1.02)**	**<0.001**	**1.01 (1.01, 1.02)**	**<0.001**	1.00 (1.00, 1.01)	0.255	1.00 (1.00, 1.01)	0.448
Falls	OR	**0.92 (0.88, 0.97)**	**<0.001**	1.00 (0.93, 1.07)	0.986	1.02 (0.95, 1.10)	0.572	0.99 (0.90, 1.09)	0.862
Gait speed	β	**0.10 (0.08, 0.12)**	**<0.001**	**-0.07 (-0.10, -0.04)**	**<0.001**	**-0.07 (-0.10, -0.04)**	**<0.001**	**0.08 (0.04, 0.12)**	**<0.001**
Lung function	β	**0.08 (0.05, 0.10)**	**<0.001**	**0.10 (0.06, 0.14)**	**<0.001**	0.00 (-0.04, 0.04)	0.931	0.03 (-0.01, 0.07)	0.215
Grip strength	β	**0.08 (0.07, 0.10)**	**<0.001**	**-0.08 (-0.11, -0.05)**	**<0.001**	**-0.14 (-0.16, -0.11)**	**<0.001**	**0.06 (0.02, 0.10)**	**0.003**
Static balance	OR	**1.22 (1.17, 1.26)**	**<0.001**	**1.09 (1.03, 1.15)**	**0.002**	**0.88 (0.83, 0.93)**	**<0.001**	**1.13 (1.05, 1.22)**	**0.001**
**Long-term physical health problems**									
Chronic health conditions	OR	**0.80 (0.77, 0.82)**	**<0.001**	**0.94 (0.90, 0.98)**	**0.004**	**1.06 (1.01, 1.11)**	**0.012**	0.96 (0.90, 1.02)	0.189
Degree of persistent pain	OR	**0.85 (0.83, 0.88)**	**<0.001**	1.00 (0.95, 1.05)	0.979	0.99 (0.94, 1.03)	0.550	**0.88 (0.82, 0.94)**	**<0.001**
**Heart health**									
Systolic blood pressure	OR	0.97 (0.94, 1.00)	0.085	1.00 (0.96, 1.05)	0.880	1.02 (0.97, 1.07)	0.480	1.00 (0.93, 1.07)	0.948
Diastolic blood pressure	OR	1.01 (0.97, 1.05)	0.654	1.05 (0.99, 1.11)	0.095	1.00 (0.95, 1.07)	0.888	0.95 (0.88, 1.03)	0.224
Pulse	β	**-0.05 (-0.06, -0.03)**	**<0.001**	**0.03 (0.00, 0.05)**	**0.037**	0.01 (-0.02, 0.03)	0.552	**-0.04 (-0.08, -0.01)**	**0.021**
**Weight**									
BMI	OR	**0.83 (0.81, 0.86)**	**<0.001**	1.01 (0.96, 1.07)	0.568	1.04 (0.99, 1.10)	0.105	1.02 (0.95, 1.10)	0.560
Waist circumference	β	**-0.11 (-0.13, -0.09)**	**<0.001**	**-0.07 (-0.10, -0.05)**	**<0.001**	0.02 (-0.01, 0.05)	0.141	0.01 (-0.02, 0.05)	0.438
**Sleep**									
Uses sleep medication	OR	**0.92 (0.88, 0.96)**	**<0.001**	**0.88 (0.82, 0.95)**	**0.001**	0.99 (0.92, 1.07)	0.845	0.96 (0.87, 1.06)	0.433
How often does not feel rested	OR	**0.89 (0.86, 0.92)**	**<0.001**	**0.93 (0.89, 0.98)**	**0.003**	**0.92 (0.88, 0.97)**	**0.001**	**0.90 (0.84, 0.96)**	**0.002**
**Subjective perceptions of health**									
Poor eyesight	OR	**0.88 (0.86, 0.91)**	**<0.001**	**0.95 (0.91, 0.99)**	**0.018**	**0.88 (0.84, 0.92)**	**<0.001**	**0.85 (0.80, 0.90)**	**<0.001**
Poor hearing	OR	**0.92 (0.90, 0.95)**	**<0.001**	**0.92 (0.88, 0.96)**	**<0.001**	**0.92 (0.88, 0.96)**	**<0.001**	**0.93 (0.88, 0.99)**	**0.017**
Perceived difficulty with balance	OR	**0.85 (0.83, 0.87)**	**<0.001**	**0.93 (0.89, 0.97)**	**0.001**	**1.08 (1.03, 1.12)**	**0.001**	0.95 (0.89, 1.00)	0.063

Note. Bold text indicates $p<0.05$. Results weighted and based on 20 imputed datasets. IRR: incidence rate ratio from negative binomial regression model. OR: odds ratio from ordered logistic regression model (binary logistic regression for falls). β: standardized coefficient from linear regression model. All tests were two-sided. No adjustments were made for multiple comparisons.

Table S6. Adjusted regression models testing longitudinal associations between leisure engagement and experiences of aging eight years later.

		Physical activities			Creative activities			Cognitive activities			Community activities		
	Coef	Coef (95% CI)	p	E (CI)	Coef (95% CI)	p	E (CI)	Coef (95% CI)	p	E (CI)	Coef (95% CI)	p	E (CI)
**Daily functioning**													
Difficulties with ADLs	IRR	**0.91 (0.87, 0.95)**	**<0.001**	1.43 (1.29)	**0.89 (0.84, 0.95)**	**<0.001**	1.50 (1.29)	0.95 (0.89, 1.02)	0.135	1.29 (1.00)	1.02 (0.94, 1.12)	0.596	1.16 (1.00)
Difficulties with IADLs	IRR	**0.95 (0.91, 0.99)**	**0.015**	1.29 (1.11)	**0.90 (0.84, 0.96)**	**0.002**	1.46 (1.25)	0.97 (0.90, 1.04)	0.399	1.21 (1.00)	1.02 (0.93, 1.11)	0.673	1.16 (1.00)
Difficulties with mobility	OR	**0.88 (0.86, 0.91)**	**<0.001**	1.32 (1.27)	0.96 (0.91, 1.00)	0.064	1.17 (1.00)	1.03 (0.99, 1.08)	0.177	1.14 (1.00)	0.99 (0.93, 1.06)	0.826	1.08 (1.00)
**Physical fitness**													
Strength	IRR	**1.02 (1.01, 1.03)**	**<0.001**	1.16 (1.11)	**1.02 (1.01, 1.03)**	**0.002**	1.16 (1.11)	1.00 (0.99, 1.01)	0.658	1.05 (1.00)	1.00 (0.99, 1.01)	0.917	1.03 (1.00)
Gross motor function	IRR	**1.01 (1.00, 1.01)**	**<0.001**	1.10 (1.07)	**1.01 (1.00, 1.01)**	**<0.001**	1.10 (1.07)	1.00 (1.00, 1.01)	0.164	1.06 (1.00)	1.00 (1.00, 1.01)	0.201	1.07 (1.00)
Fine motor function	IRR	**1.00 (1.00, 1.01)**	**<0.001**	1.08 (1.05)	**1.01 (1.00, 1.01)**	**<0.001**	1.09 (1.06)	1.00 (1.00, 1.01)	0.139	1.06 (1.00)	1.00 (0.99, 1.00)	0.219	1.06 (1.00)
Falls	OR	**0.95 (0.91, 1.00)**	**0.046**	1.19 (1.02)	1.02 (0.94, 1.09)	0.676	1.10 (1.00)	1.00 (0.93, 1.09)	0.925	1.05 (1.00)	0.99 (0.89, 1.09)	0.791	1.09 (1.00)
Gait speed	β	**0.03 (0.01, 0.05)**	**0.002**	1.13 (1.07)	**0.07 (0.03, 0.10)**	**<0.001**	1.11 (1.04)	0.03 (0.00, 0.06)	0.092	1.12 (1.06)	0.00 (-0.04, 0.03)	0.957	1.06 (1.00)
Lung function	β	**0.01 (0.00, 0.02)**	**0.003**	1.20 (1.11)	0.01 (0.00, 0.03)	0.149	1.32 (1.20)	0.01 (0.00, 0.03)	0.119	1.19 (1.00)	0.00 (-0.02, 0.02)	0.707	1.03 (1.00)
Grip strength	β	**0.01 (0.01, 0.02)**	**0.001**	1.13 (1.08)	0.01 (0.00, 0.03)	0.077	1.12 (1.00)	0.00 (-0.01, 0.01)	0.925	1.03 (1.00)	-0.01 (-0.03, 0.01)	0.184	1.12 (1.00)
Static balance	OR	**1.13 (1.09, 1.17)**	**<0.001**	1.32 (1.26)	**1.09 (1.03, 1.16)**	**0.003**	1.32 (1.14)	0.97 (0.91, 1.03)	0.339	1.14 (1.00)	1.06 (0.98, 1.15)	0.165	1.20 (1.00)
**Long-term physical health problems**													
Chronic health conditions	OR	**0.90 (0.87, 0.93)**	**<0.001**	1.29 (1.23)	0.97 (0.93, 1.02)	0.209	1.14 (1.00)	1.01 (0.96, 1.06)	0.626	1.08 (1.00)	**1.09 (1.03, 1.17)**	**0.005**	1.16 (1.00)
Degree of persistent pain	OR	**0.95 (0.92, 0.98)**	**0.001**	1.19 (1.11)	1.00 (0.95, 1.05)	0.974	1.02 (1.00)	0.97 (0.92, 1.02)	0.287	1.14 (1.00)	0.96 (0.89, 1.03)	0.265	1.17 (1.00)
**Heart health**													
Systolic blood pressure	OR	1.00 (0.97, 1.04)	0.787	1.05 (1.00)	1.03 (0.98, 1.09)	0.262	1.14 (1.00)	1.02 (0.97, 1.08)	0.411	1.11 (1.00)	1.04 (0.97, 1.11)	0.319	1.16 (1.00)
Diastolic blood pressure	OR	1.02 (0.98, 1.07)	0.248	1.11 (1.00)	1.06 (1.00, 1.13)	0.054	1.21 (1.00)	1.02 (0.96, 1.09)	0.463	1.11 (1.00)	0.98 (0.90, 1.07)	0.681	1.11 (1.00)
Pulse	β	-0.01 (-0.02, 0.01)	0.339	1.09 (1.00)	0.02 (-0.01, 0.04)	0.215	1.14 (1.00)	0.02 (-0.01, 0.04)	0.210	1.14 (1.00)	-0.02 (-0.05, 0.01)	0.245	1.16 (1.00)
**Weight**													
BMI	OR	**0.95 (0.91, 0.99)**	**0.010**	1.19 (1.08)	1.05 (0.98, 1.12)	0.154	1.18 (1.00)	1.03 (0.97, 1.11)	0.318	1.14 (1.00)	1.02 (0.94, 1.12)	0.615	1.11 (1.00)
Waist circumference	β	0.00 (-0.01, 0.01)	0.569	1.06 (1.00)	0.00 (-0.01, 0.02)	0.827	1.04 (1.00)	0.00 (-0.01, 0.02)	0.567	1.07 (1.00)	0.01 (-0.01, 0.03)	0.536	1.09 (1.00)
**Sleep**													
Uses sleep medication	OR	0.97 (0.93, 1.02)	0.251	1.21 (1.00)	**0.84 (0.78, 0.91)**	**<0.001**	1.67 (1.43)	0.96 (0.89, 1.04)	0.350	1.25 (1.00)	1.04 (0.94, 1.16)	0.423	1.24 (1.00)
How often does not feel rested	OR	**0.95 (0.92, 0.98)**	**0.002**	1.19 (1.11)	0.97 (0.92, 1.02)	0.237	1.14 (1.00)	**0.93 (0.88, 0.98)**	**0.004**	1.23 (1.11)	0.97 (0.90, 1.04)	0.365	1.14 (1.00)
**Subjective perceptions of health**													
Poor eyesight	OR	**0.95 (0.93, 0.98)**	**0.002**	1.19 (1.11)	0.97 (0.93, 1.02)	0.232	1.14 (1.00)	**0.94 (0.90, 0.99)**	**0.011**	1.21 (1.08)	0.95 (0.89, 1.01)	0.120	1.19 (1.00)
Poor hearing	OR	**0.96 (0.93, 0.99)**	**0.004**	1.17 (1.08)	0.97 (0.93, 1.02)	0.230	1.14 (1.00)	0.97 (0.92, 1.01)	0.150	1.14 (1.00)	0.96 (0.90, 1.02)	0.179	1.16 (1.00)
Perceived difficulty with balance	OR	**0.91 (0.89, 0.94)**	**<0.001**	1.27 (1.21)	**0.94 (0.90, 0.99)**	**0.011**	1.21 (1.08)	1.02 (0.97, 1.07)	0.532	1.11 (1.00)	1.02 (0.95, 1.09)	0.591	1.11 (1.00)

Note. All models adjusted for age, gender, race/ethnicity, marital status, education, employment, pension status, household income, assets, household size, neighborhood safety, neighborhood physical disorder, neighborhood social cohesion, and the baseline measure of the outcome. Bold text indicates $p<0.05$. Results weighted and based on 20 imputed datasets. IRR: incidence rate ratio from negative binomial regression model. OR: odds ratio from ordered logistic regression model (binary logistic regression for falls and sleep medication). β: standardized coefficient from linear regression model. All tests were two-sided. No adjustments were made for multiple comparisons.

Table S7. Unadjusted regression models testing concurrent associations between leisure engagement and experiences of aging.

		**Physical activities**		**Creative activities**		**Cognitive activities**		**Community activities**	
	Coef	Coef (95% CI)	p	Coef (95% CI)	p	Coef (95% CI)	p	Coef (95% CI)	p
**Daily functioning**									
Difficulties with ADLs	IRR	**0.75 (0.71, 0.79)**	**<0.001**	**0.79 (0.73, 0.87)**	**<0.001**	0.95 (0.87, 1.03)	0.187	**0.86 (0.74, 0.99)**	**0.035**
Difficulties with IADLs	IRR	**0.82 (0.77, 0.87)**	**<0.001**	**0.71 (0.64, 0.79)**	**<0.001**	**0.89 (0.81, 0.98)**	**0.022**	**0.79 (0.67, 0.93)**	**0.004**
Difficulties with mobility	OR	**0.71 (0.69, 0.73)**	**<0.001**	**0.93 (0.89, 0.97)**	**0.001**	**1.06 (1.02, 1.11)**	**0.009**	**0.87 (0.82, 0.93)**	**<0.001**
**Physical fitness**									
Strength	IRR	**1.06 (1.05, 1.07)**	**<0.001**	**1.03 (1.01, 1.04)**	**<0.001**	**0.99 (0.98, 1.00)**	**0.038**	**1.04 (1.03, 1.05)**	**<0.001**
Gross motor function	IRR	**1.02 (1.02, 1.02)**	**<0.001**	**1.01 (1.01, 1.02)**	**<0.001**	1.00 (1.00, 1.01)	0.590	**1.01 (1.00, 1.01)**	**0.002**
Fine motor function	IRR	**1.01 (1.01, 1.01)**	**<0.001**	**1.01 (1.00, 1.01)**	**<0.001**	1.00 (1.00, 1.01)	0.185	**1.01 (1.00, 1.01)**	**0.001**
Falls	OR	**0.89 (0.85, 0.93)**	**<0.001**	1.04 (0.97, 1.12)	0.239	0.98 (0.91, 1.05)	0.590	1.07 (0.97, 1.18)	0.187
Gait speed	β	**0.09 (0.08, 0.11)**	**<0.001**	**-0.09 (-0.11, -0.06)**	**<0.001**	**-0.08 (-0.11, -0.05)**	**<0.001**	**0.09 (0.05, 0.12)**	**<0.001**
Lung function	β	**0.06 (0.04, 0.08)**	**<0.001**	**0.06 (0.03, 0.10)**	**0.001**	0.00 (-0.03, 0.03)	0.965	0.04 (0.00, 0.08)	0.055
Grip strength	β	**0.07 (0.05, 0.09)**	**<0.001**	**-0.09 (-0.12, -0.06)**	**<0.001**	**-0.13 (-0.16, -0.10)**	**<0.001**	**0.08 (0.04, 0.12)**	**<0.001**
Static balance	OR	**1.20 (1.15, 1.25)**	**<0.001**	**1.11 (1.04, 1.19)**	**0.002**	**0.91 (0.86, 0.98)**	**0.009**	**1.14 (1.04, 1.26)**	**0.006**
**Long-term physical health problems**									
Chronic health conditions	OR	**0.81 (0.79, 0.83)**	**<0.001**	**0.95 (0.91, 0.99)**	**0.023**	**1.08 (1.03, 1.13)**	**0.002**	**0.90 (0.85, 0.96)**	**0.002**
Degree of persistent pain	OR	**0.80 (0.78, 0.83)**	**<0.001**	0.97 (0.92, 1.02)	0.227	1.02 (0.97, 1.07)	0.451	**0.85 (0.79, 0.91)**	**<0.001**
**Heart health**									
Systolic blood pressure	OR	**0.95 (0.92, 0.99)**	**0.004**	1.00 (0.96, 1.05)	0.879	1.02 (0.97, 1.08)	0.360	**0.93 (0.87, 0.99)**	**0.028**
Diastolic blood pressure	OR	**0.95 (0.92, 0.98)**	**0.003**	1.04 (0.99, 1.10)	0.137	1.03 (0.97, 1.08)	0.349	**0.93 (0.86, 1.00)**	**0.039**
Pulse	β	**-0.08 (-0.10, -0.07)**	**<0.001**	0.02 (-0.01, 0.05)	0.223	-0.01 (-0.04, 0.02)	0.486	-0.03 (-0.07, 0.01)	0.179
**Weight**									
BMI	OR	**0.79 (0.77, 0.82)**	**<0.001**	0.98 (0.93, 1.04)	0.543	**1.07 (1.02, 1.12)**	**0.011**	1.01 (0.94, 1.08)	0.742
Waist circumference	β	**-0.15 (-0.16, -0.13)**	**<0.001**	**-0.08 (-0.11, -0.06)**	**<0.001**	**0.03 (0.01, 0.06)**	**0.010**	0.01 (-0.03, 0.04)	0.655
**Sleep**									
Uses sleep medication	OR	**0.91 (0.88, 0.95)**	**<0.001**	1.03 (0.97, 1.09)	0.310	**1.08 (1.02, 1.15)**	**0.010**	0.93 (0.86, 1.01)	0.084
How often does not feel rested	OR	**0.87 (0.84, 0.90)**	**<0.001**	**0.91 (0.86, 0.95)**	**<0.001**	0.99 (0.95, 1.04)	0.812	**0.89 (0.83, 0.95)**	**0.001**
**Subjective perceptions of health**									
Poor eyesight	OR	**0.89 (0.87, 0.92)**	**<0.001**	**0.93 (0.89, 0.97)**	**0.001**	**0.88 (0.84, 0.92)**	**<0.001**	**0.85 (0.80, 0.91)**	**<0.001**
Poor hearing	OR	**0.93 (0.91, 0.96)**	**<0.001**	**0.92 (0.88, 0.96)**	**<0.001**	**0.92 (0.88, 0.96)**	**<0.001**	**0.93 (0.88, 0.99)**	**0.023**
Perceived difficulty with balance	OR	**0.86 (0.83, 0.88)**	**<0.001**	**0.93 (0.89, 0.97)**	**0.002**	**1.05 (1.00, 1.10)**	**0.045**	**0.88 (0.83, 0.94)**	**<0.001**

Note. Bold text indicates $p<0.05$. Results weighted and based on 20 imputed datasets. IRR: incidence rate ratio from negative binomial regression model. OR: odds ratio from ordered logistic regression model (binary logistic regression for falls and sleep medication). β: standardized coefficient from linear regression model. All tests were two-sided. No adjustments were made for multiple comparisons.

Table S8. Adjusted regression models testing concurrent associations between leisure engagement and experiences of aging.

		Physical activities		Creative activities		Cognitive activities		Community activities	
	Coef	Coef (95% CI)	p	Coef (95% CI)	p	Coef (95% CI)	p	Coef (95% CI)	p
**Daily functioning**									
Difficulties with ADLs	IRR	**0.79 (0.75, 0.84)**	**<0.001**	**0.81 (0.75, 0.89)**	**<0.001**	0.98 (0.90, 1.07)	0.675	0.94 (0.83, 1.06)	0.287
Difficulties with IADLs	IRR	**0.88 (0.83, 0.94)**	**<0.001**	**0.69 (0.62, 0.77)**	**<0.001**	0.93 (0.84, 1.03)	0.143	0.93 (0.81, 1.07)	0.314
Difficulties with mobility	OR	**0.73 (0.71, 0.76)**	**<0.001**	**0.88 (0.84, 0.92)**	**<0.001**	1.04 (0.99, 1.09)	0.125	0.96 (0.90, 1.02)	0.179
**Physical fitness**									
Strength	IRR	**1.04 (1.04, 1.05)**	**<0.001**	**1.04 (1.03, 1.06)**	**<0.001**	1.00 (0.98, 1.01)	0.367	**1.02 (1.01, 1.03)**	**0.006**
Gross motor function	IRR	**1.02 (1.01, 1.02)**	**<0.001**	**1.01 (1.01, 1.02)**	**<0.001**	1.00 (1.00, 1.00)	0.727	1.00 (1.00, 1.01)	0.137
Fine motor function	IRR	**1.01 (1.00, 1.01)**	**<0.001**	**1.01 (1.01, 1.01)**	**<0.001**	1.00 (1.00, 1.00)	0.666	1.00 (1.00, 1.01)	0.086
Falls	OR	**0.90 (0.86, 0.95)**	**<0.001**	1.04 (0.96, 1.12)	0.327	0.93 (0.86, 1.01)	0.077	1.07 (0.97, 1.19)	0.185
Gait speed	β	**0.04 (0.02, 0.06)**	**<0.001**	**0.06 (0.02, 0.10)**	**0.003**	0.00 (-0.03, 0.03)	0.973	0.03 (-0.01, 0.06)	0.178
Lung function	β	**0.03 (0.02, 0.04)**	**<0.001**	0.01 (-0.01, 0.03)	0.178	0.02 (0.00, 0.04)	0.117	0.02 (-0.01, 0.05)	0.325
Grip strength	β	0.00 (-0.01, 0.01)	0.505	**0.05 (0.04, 0.07)**	**<0.001**	0.02 (0.00, 0.04)	0.074	0.01 (-0.01, 0.04)	0.361
Static balance	OR	1.14 (1.09, 1.19)	<0.001	**1.14 (1.07, 1.21)**	**<0.001**	0.98 (0.91, 1.05)	0.492	1.07 (0.97, 1.18)	0.178
**Long-term physical health problems**									
Chronic health conditions	OR	**0.84 (0.82, 0.87)**	**<0.001**	**0.93 (0.89, 0.97)**	**0.002**	1.04 (0.99, 1.09)	0.104	0.96 (0.89, 1.02)	0.168
Degree of persistent pain	OR	**0.84 (0.81, 0.87)**	**<0.001**	**0.93 (0.88, 0.98)**	**0.006**	1.04 (0.98, 1.09)	0.167	0.94 (0.88, 1.02)	0.123
**Heart health**									
Systolic blood pressure	OR	0.97 (0.94, 1.00)	0.076	**1.08 (1.02, 1.13)**	**0.005**	1.04 (0.98, 1.10)	0.175	**0.92 (0.86, 0.98)**	**0.016**
Diastolic blood pressure	OR	**0.95 (0.92, 0.98)**	**0.003**	**1.07 (1.01, 1.13)**	**0.026**	**1.08 (1.02, 1.14)**	**0.012**	0.93 (0.86, 1.00)	0.051
Pulse	β	**-0.08 (-0.10, -0.06)**	**<0.001**	0.02 (-0.01, 0.05)	0.150	0.00 (-0.02, 0.03)	0.738	-0.01 (-0.06, 0.03)	0.494
**Weight**									
BMI	OR	**0.80 (0.77, 0.83)**	**<0.001**	1.02 (0.96, 1.07)	0.582	**1.15 (1.09, 1.21)**	**<0.001**	1.07 (0.99, 1.14)	0.082
Waist circumference	β	**-0.15 (-0.16, -0.13)**	**<0.001**	**-0.03 (-0.06, -0.01)**	**0.013**	**0.09 (0.06, 0.12)**	**<0.001**	0.02 (-0.02, 0.05)	0.362
**Sleep**									
Uses sleep medication	OR	**0.93 (0.90, 0.97)**	**0.001**	0.95 (0.89, 1.01)	0.081	1.01 (0.95, 1.08)	0.695	0.97 (0.89, 1.06)	0.484
How often does not feel rested	OR	**0.88 (0.86, 0.91)**	**<0.001**	**0.89 (0.85, 0.94)**	**<0.001**	1.02 (0.97, 1.07)	0.426	**0.93 (0.87, 1.00)**	**0.050**
**Subjective perceptions of health**									
Poor eyesight	OR	**0.94 (0.91, 0.97)**	**<0.001**	**0.93 (0.89, 0.98)**	**0.004**	**0.93 (0.88, 0.97)**	**0.002**	**0.93 (0.88, 1.00)**	**0.041**
Poor hearing	OR	**0.95 (0.92, 0.97)**	**<0.001**	0.96 (0.91, 1.00)	0.056	0.97 (0.93, 1.02)	0.223	0.97 (0.91, 1.03)	0.338
Perceived difficulty with balance	OR	**0.90 (0.87, 0.92)**	**<0.001**	**0.89 (0.85, 0.93)**	**<0.001**	0.99 (0.94, 1.04)	0.802	**0.93 (0.88, 0.99)**	**0.034**

Note. All models adjusted for age, gender, race/ethnicity, marital status, education, employment, pension status, household income, assets, household size, neighborhood safety, neighborhood physical disorder, and neighborhood social cohesion. Bold text indicates $p<0.05$. Results weighted and based on 20 imputed datasets. IRR: incidence rate ratio from negative binomial regression model. OR: odds ratio from ordered logistic regression model (binary logistic regression for falls and sleep medication).
β: standardized coefficient from linear regression model. All tests were two-sided. No adjustments were made for multiple comparisons.

Table S9. Adjusted regression models testing longitudinal associations between leisure engagement and experiences of aging eight years later, additionally adjusted for health and health behavior covariates.

		Physical activities			Creative activities			Cognitive activities			Community activities		
	Coef	Coef (95% CI)	p	E (CI)	Coef (95% CI)	p	E (CI)	Coef (95% CI)	p	E (CI)	Coef (95% CI)	p	E (CI)
**Daily functioning**													
Difficulties with ADLs	IRR	**0.95 (0.91, 1.00)**	**0.029**	1.28 (1.08)	**0.91 (0.85, 0.96)**	**0.001**	1.43 (1.25)	0.98 (0.92, 1.04)	0.540	1.17 (1.00)	1.06 (0.98, 1.15)	0.169	1.31 (1.00)
Difficulties with IADLs	IRR	1.00 (0.96, 1.05)	0.926	1.00 (1.00)	**0.92 (0.86, 0.98)**	**0.007**	1.39 (1.17)	1.02 (0.95, 1.09)	0.657	1.16 (1.00)	1.05 (0.97, 1.15)	0.229	1.28 (1.00)
Difficulties with mobility	OR	**0.91 (0.88, 0.94)**	**<0.001**	1.27 (1.21)	0.96 (0.92, 1.01)	0.128	1.17 (1.00)	1.04 (0.99, 1.09)	0.128	1.16 (1.00)	1.02 (0.96, 1.09)	0.532	1.11 (1.00)
**Physical fitness**													
Strength	IRR	**1.01 (1.00, 1.02)**	**0.002**	1.11 (1.07)	**1.01 (1.00, 1.02)**	**0.010**	1.13 (1.06)	1.00 (0.99, 1.01)	0.902	1.00 (1.00)	1.00 (0.98, 1.01)	0.588	1.00 (1.00)
Gross motor function	IRR	**1.00 (1.00, 1.01)**	**0.002**	1.08 (1.04)	**1.01 (1.00, 1.01)**	**0.001**	1.10 (1.06)	1.00 (1.00, 1.01)	0.513	1.00 (1.00)	1.00 (1.00, 1.01)	0.555	1.00 (1.00)
Fine motor function	IRR	1.00 (1.00, 1.00)	0.093	1.00 (1.00)	**1.01 (1.00, 1.01)**	**0.005**	1.08 (1.04)	1.00 (1.00, 1.01)	0.292	1.00 (1.00)	1.00 (0.99, 1.00)	0.090	1.00 (1.00)
Falls	OR	0.97 (0.93, 1.02)	0.285	1.14 (1.00)	1.04 (0.96, 1.12)	0.322	1.16 (1.00)	1.02 (0.94, 1.10)	0.624	1.11 (1.00)	0.99 (0.90, 1.10)	0.853	1.08 (1.00)
Gait speed	β	0.01 (0.00, 0.02)	0.246	1.08 (1.00)	0.01 (0.00, 0.03)	0.137	1.11 (1.00)	0.01 (0.00, 0.03)	0.106	1.12 (1.00)	-0.01 (-0.03, 0.01)	0.163	1.13 (1.00)
Lung function	β	**0.02 (0.00, 0.04)**	**0.032**	1.16 (1.04)	**0.06 (0.03, 0.10)**	**<0.001**	1.31 (1.19)	0.01 (-0.02, 0.05)	0.357	1.13 (1.00)	0.00 (-0.04, 0.03)	0.819	1.07 (1.00)
Grip strength	β	**0.01 (0.00, 0.02)**	**0.021**	1.11 (1.04)	0.01 (0.00, 0.03)	0.110	1.11 (1.00)	0.00 (-0.01, 0.01)	0.964	1.02 (1.00)	-0.01 (-0.03, 0.00)	0.132	1.12 (1.00)
Static balance	OR	**1.08 (1.04, 1.12)**	**<0.001**	1.24 (1.16)	**1.07 (1.01, 1.13)**	**0.028**	1.22 (1.08)	0.96 (0.90, 1.02)	0.150	1.17 (1.00)	1.04 (0.96, 1.14)	0.327	1.16 (1.00)
**Long-term physical health problems**													
Chronic health conditions	OR	**0.93 (0.90, 0.96)**	**<0.001**	1.23 (1.17)	0.98 (0.94, 1.03)	0.432	1.11 (1.00)	1.01 (0.96, 1.06)	0.685	1.08 (1.00)	**1.12 (1.05, 1.20)**	**<0.001**	1.31 (1.18)
Degree of persistent pain	OR	0.98 (0.95, 1.02)	0.301	1.11 (1.00)	1.02 (0.97, 1.08)	0.370	1.11 (1.00)	0.97 (0.92, 1.03)	0.328	1.14 (1.00)	0.97 (0.90, 1.04)	0.410	1.14 (1.00)
**Heart health**													
Systolic blood pressure	OR	1.00 (0.97, 1.04)	0.772	1.00 (1.00)	1.03 (0.97, 1.08)	0.324	1.14 (1.00)	1.02 (0.96, 1.08)	0.506	1.11 (1.00)	1.04 (0.97, 1.11)	0.332	1.16 (1.00)
Diastolic blood pressure	OR	1.02 (0.97, 1.06)	0.450	1.11 (1.00)	1.06 (1.00, 1.13)	0.071	1.20 (1.00)	1.03 (0.96, 1.09)	0.435	1.14 (1.00)	0.98 (0.89, 1.06)	0.574	1.11 (1.00)
Pulse	β	0.00 (-0.02, 0.01)	0.542	1.07 (1.00)	0.02 (-0.01, 0.04)	0.218	1.14 (1.00)	0.02 (-0.01, 0.04)	0.223	1.13 (1.00)	-0.02 (-0.05, 0.02)	0.294	1.15 (1.00)
**Weight**													
BMI	OR	0.96 (0.92, 1.00)	0.081	1.17 (1.00)	1.06 (0.99, 1.14)	0.080	1.20 (1.00)	1.03 (0.96, 1.11)	0.354	1.14 (1.00)	1.03 (0.94, 1.13)	0.511	1.14 (1.00)
Waist circumference	β	0.01 (0.00, 0.02)	0.167	1.09 (1.00)	0.00 (-0.01, 0.02)	0.701	1.06 (1.00)	0.00 (-0.01, 0.02)	0.689	1.06 (1.00)	0.01 (-0.01, 0.03)	0.467	1.10 (1.00)
**Sleep**													
Uses sleep medication	OR	1.04 (0.99, 1.09)	0.157	1.24 (1.00)	**0.88 (0.81, 0.95)**	**0.001**	1.53 (1.29)	0.96 (0.89, 1.04)	0.344	1.25 (1.00)	1.09 (0.98, 1.22)	0.104	1.40 (1.00)
How often does not feel rested	OR	0.98 (0.95, 1.02)	0.343	1.11 (1.00)	0.98 (0.94, 1.04)	0.549	1.11 (1.00)	**0.93 (0.89, 0.98)**	**0.011**	1.23 (1.11)	0.98 (0.92, 1.06)	0.666	1.11 (1.00)
**Subjective perceptions of health**													
Poor eyesight	OR	0.99 (0.96, 1.02)	0.436	1.08 (1.00)	0.98 (0.93, 1.03)	0.361	1.11 (1.00)	**0.94 (0.89, 0.98)**	**0.007**	1.21 (1.11)	0.97 (0.91, 1.03)	0.313	1.14 (1.00)
Poor hearing	OR	0.97 (0.95, 1.00)	0.080	1.14 (1.00)	0.98 (0.94, 1.03)	0.454	1.11 (1.00)	0.97 (0.93, 1.02)	0.213	1.14 (1.00)	0.97 (0.91, 1.03)	0.295	1.14 (1.00)
Perceived difficulty with balance	OR	**0.95 (0.92, 0.98)**	**<0.001**	1.19 (1.11)	0.96 (0.91, 1.00)	0.065	1.17 (1.00)	1.02 (0.97, 1.07)	0.395	1.11 (1.00)	1.03 (0.97, 1.10)	0.330	1.14 (1.00)

Note. All models adjusted for age, gender, race/ethnicity, marital status, education, employment, pension status, household income, assets, household size, neighborhood safety, neighborhood physical disorder, neighborhood social cohesion, cognition, depressive symptoms, prescription medication, psychiatric problems, self-rated health, and the outcome measured at baseline, as well as alcohol use and smoking measured at the wave prior to baseline. Bold text indicates $p<0.05$. Results weighted and based on 20 imputed datasets. IRR: incidence rate ratio from negative binomial regression model. OR: odds ratio from ordered logistic regression model (binary logistic regression for falls and sleep medication). β: standardized coefficient from linear regression model. All tests were two-sided. No adjustments were made for multiple comparisons.

Table S10. Adjusted regression models testing longitudinal associations between leisure engagement and experiences of aging eight years later, with the baseline measure of outcome omitted.

		**Physical activities**		**Creative activities**		**Cognitive activities**		**Community activities**	
	Coef	Coef (95% CI)	p	Coef (95% CI)	p	Coef (95% CI)	p	Coef (95% CI)	p
**Daily functioning**									
Difficulties with ADLs	IRR	**0.86 (0.82, 0.89)**	**<0.001**	**0.84 (0.79, 0.90)**	**<0.001**	0.95 (0.89, 1.01)	0.114	1.03 (0.94, 1.12)	0.559
Difficulties with IADLs	IRR	**0.91 (0.87, 0.96)**	**<0.001**	**0.83 (0.77, 0.88)**	**<0.001**	0.95 (0.88, 1.02)	0.149	1.01 (0.92, 1.11)	0.800
Difficulties with mobility	OR	**0.77 (0.75, 0.80)**	**<0.001**	**0.90 (0.86, 0.94)**	**<0.001**	1.05 (1.00, 1.10)	0.056	0.98 (0.92, 1.04)	0.440
**Physical fitness**									
Strength	IRR	**1.05 (1.04, 1.05)**	**<0.001**	**1.04 (1.03, 1.05)**	**<0.001**	1.00 (0.99, 1.01)	0.899	1.01 (0.99, 1.03)	0.232
Gross motor function	IRR	**1.02 (1.02, 1.02)**	**<0.001**	**1.02 (1.01, 1.02)**	**<0.001**	1.00 (1.00, 1.01)	0.245	1.01 (1.00, 1.01)	0.056
Fine motor function	IRR	**1.01 (1.01, 1.01)**	**<0.001**	**1.01 (1.01, 1.02)**	**<0.001**	1.00 (1.00, 1.01)	0.137	1.00 (0.99, 1.00)	0.717
Falls	OR	**0.93 (0.89, 0.98)**	**0.004**	1.02 (0.95, 1.10)	0.526	0.99 (0.92, 1.07)	0.746	1.00 (0.91, 1.11)	0.970
Gait speed	β	**0.05 (0.03, 0.07)**	**<0.001**	**0.09 (0.05, 0.13)**	**<0.001**	0.03 (-0.01, 0.06)	0.114	0.01 (-0.03, 0.05)	0.611
Lung function	β	**0.04 (0.02, 0.05)**	**<0.001**	**0.02 (0.00, 0.04)**	**0.042**	**0.03 (0.00, 0.05)**	**0.021**	0.01 (-0.02, 0.04)	0.639
Grip strength	β	**0.02 (0.01, 0.03)**	**0.002**	**0.05 (0.03, 0.07)**	**<0.001**	0.01 (-0.01, 0.03)	0.182	-0.01 (-0.03, 0.02)	0.687
Static balance	OR	**1.15 (1.11, 1.20)**	**<0.001**	**1.12 (1.06, 1.19)**	**<0.001**	0.96 (0.91, 1.03)	0.243	1.07 (0.99, 1.16)	0.094
**Long-term physical health problems**									
Chronic health conditions	OR	**0.83 (0.80, 0.85)**	**<0.001**	**0.93 (0.89, 0.97)**	**0.002**	1.05 (1.00, 1.10)	0.064	1.02 (0.95, 1.08)	0.595
Degree of persistent pain	OR	**0.88 (0.86, 0.91)**	**<0.001**	0.97 (0.92, 1.02)	0.254	0.99 (0.94, 1.04)	0.600	0.94 (0.88, 1.01)	0.080
**Heart health**									
Systolic blood pressure	OR	0.99 (0.96, 1.03)	0.737	1.05 (1.00, 1.10)	0.065	1.03 (0.98, 1.09)	0.213	1.01 (0.94, 1.08)	0.761
Diastolic blood pressure	OR	1.01 (0.97, 1.05)	0.739	**1.08 (1.02, 1.15)**	**0.012**	1.05 (0.98, 1.12)	0.145	0.96 (0.88, 1.04)	0.343
Pulse	β	**-0.04 (-0.06, -0.03)**	**<0.001**	0.02 (0.00, 0.05)	0.069	0.02 (-0.01, 0.04)	0.198	-0.03 (-0.06, 0.01)	0.169
**Weight**									
BMI	OR	**0.83 (0.80, 0.85)**	**<0.001**	1.04 (0.99, 1.10)	0.110	**1.14 (1.07, 1.20)**	**<0.001**	1.06 (0.99, 1.14)	0.110
Waist circumference	β	**-0.12 (-0.13, -0.10)**	**<0.001**	-0.02 (-0.05, 0.00)	0.056	**0.08 (0.05, 0.10)**	**<0.001**	0.02 (-0.02, 0.05)	0.280
**Sleep**									
Uses sleep medication	OR	**0.95 (0.91, 0.99)**	**0.015**	**0.84 (0.78, 0.91)**	**<0.001**	0.97 (0.90, 1.05)	0.463	1.02 (0.93, 1.13)	0.634
How often does not feel rested	OR	**0.91 (0.88, 0.94)**	**<0.001**	**0.93 (0.89, 0.98)**	**0.004**	**0.95 (0.90, 1.00)**	**0.033**	0.95 (0.88, 1.01)	0.109
**Subjective perceptions of health**									
Poor eyesight	OR	**0.93 (0.90, 0.96)**	**<0.001**	**0.95 (0.91, 1.00)**	**0.044**	**0.92 (0.87, 0.96)**	**<0.001**	**0.93 (0.87, 0.99)**	**0.021**
Poor hearing	OR	**0.94 (0.91, 0.96)**	**<0.001**	**0.95 (0.91, 0.99)**	**0.028**	**0.95 (0.91, 1.00)**	**0.046**	0.96 (0.90, 1.02)	0.165
Perceived difficulty with balance	OR	**0.88 (0.86, 0.91)**	**<0.001**	**0.90 (0.85, 0.94)**	**<0.001**	1.01 (0.96, 1.06)	0.658	0.99 (0.93, 1.05)	0.702

Note. All models adjusted for age, gender, race/ethnicity, marital status, education, employment, pension status, household income, assets, household size, neighborhood safety, neighborhood physical disorder, and neighborhood social cohesion. Bold text indicates $p<0.05$. Results weighted and based on 20 imputed datasets. IRR: incidence rate ratio from negative binomial regression model. OR: odds ratio from ordered logistic regression model (binary logistic regression for falls and sleep medication).
β: standardized coefficient from linear regression model. All tests were two-sided. No adjustments were made for multiple comparisons.

Table S11A. Adjusted regression models testing longitudinal associations between different levels of leisure engagement and experiences of aging eight years later (part A).

		**Physical activities**				**Creative activities**			
		**Monthly**		**Weekly**		**Monthly**		**Weekly**	
	Coef	Coef (95% CI)	p	Coef (95% CI)	p	Coef (95% CI)	p	Coef (95% CI)	p
**Daily functioning**									
Difficulties with ADLs	IRR	**0.76 (0.64, 0.91)**	**0.003**	**0.64 (0.54, 0.77)**	**<0.001**	**0.74 (0.61, 0.89)**	**0.002**	**0.64 (0.51, 0.81)**	**<0.001**
Difficulties with IADLs	IRR	**0.75 (0.63, 0.91)**	**0.003**	**0.72 (0.59, 0.87)**	**0.001**	**0.80 (0.65, 0.98)**	**0.030**	**0.63 (0.49, 0.81)**	**<0.001**
Difficulties with mobility	OR	**0.83 (0.71, 0.96)**	**0.014**	**0.63 (0.54, 0.73)**	**<0.001**	0.97 (0.81, 1.15)	0.717	0.92 (0.76, 1.13)	0.435
**Physical fitness**									
Strength	IRR	**1.06 (1.02, 1.10)**	**0.007**	**1.09 (1.05, 1.14)**	**<0.001**	**1.08 (1.03, 1.13)**	**0.001**	**1.09 (1.04, 1.15)**	**0.001**
Gross motor function	IRR	**1.02 (1.00, 1.04)**	**0.020**	**1.04 (1.02, 1.05)**	**<0.001**	**1.04 (1.02, 1.06)**	**0.001**	**1.05 (1.02, 1.07)**	**<0.001**
Fine motor function	IRR	**1.02 (1.01, 1.04)**	**0.003**	**1.03 (1.01, 1.04)**	**<0.001**	**1.03 (1.01, 1.05)**	**0.008**	**1.04 (1.01, 1.06)**	**0.001**
Falls	OR	0.85 (0.67, 1.07)	0.156	**0.72 (0.58, 0.91)**	**0.006**	0.98 (0.74, 1.31)	0.894	1.08 (0.78, 1.48)	0.648
Gait speed	β	0.09 (-0.03, 0.22)	0.137	**0.13 (0.02, 0.24)**	**0.022**	**0.34 (0.16, 0.53)**	**<0.001**	**0.40 (0.21, 0.59)**	**<0.001**
Lung function	β	0.03 (-0.02, 0.08)	0.212	**0.07 (0.03, 0.12)**	**0.002**	-0.01 (-0.08, 0.05)	0.713	0.01 (-0.06, 0.08)	0.821
Grip strength	β	0.02 (-0.02, 0.07)	0.332	**0.08 (0.03, 0.12)**	**<0.001**	0.04 (-0.02, 0.10)	0.152	0.06 (-0.01, 0.12)	0.088
Static balance	OR	**1.26 (1.05, 1.50)**	**0.011**	**1.70 (1.43, 2.03)**	**<0.001**	1.19 (0.94, 1.51)	0.147	**1.39 (1.07, 1.80)**	**0.013**
**Long-term physical health problems**									
Chronic health conditions	OR	1.02 (0.87, 1.19)	0.843	**0.73 (0.63, 0.85)**	**<0.001**	1.02 (0.85, 1.22)	0.855	0.95 (0.78, 1.16)	0.588
Degree of persistent pain	OR	0.89 (0.76, 1.04)	0.141	**0.82 (0.71, 0.95)**	**0.009**	1.07 (0.89, 1.29)	0.481	1.00 (0.81, 1.24)	0.987
**Heart health**									
Systolic blood pressure	OR	0.88 (0.74, 1.04)	0.123	0.95 (0.81, 1.12)	0.544	1.02 (0.83, 1.25)	0.846	1.11 (0.88, 1.40)	0.366
Diastolic blood pressure	OR	0.91 (0.75, 1.12)	0.379	1.03 (0.86, 1.25)	0.729	1.09 (0.87, 1.36)	0.460	1.23 (0.95, 1.58)	0.111
Pulse	β	-0.03 (-0.11, 0.05)	0.512	-0.04 (-0.11, 0.04)	0.335	0.02 (-0.08, 0.11)	0.749	0.05 (-0.06, 0.15)	0.370
**Weight**									
BMI	OR	0.96 (0.77, 1.18)	0.671	**0.81 (0.66, 1.00)**	**0.047**	0.96 (0.74, 1.25)	0.783	1.07 (0.80, 1.43)	0.661
Waist circumference	β	0.04 (-0.01, 0.10)	0.100	0.03 (-0.02, 0.08)	0.302	0.03 (-0.03, 0.09)	0.323	0.02 (-0.05, 0.09)	0.519
**Sleep**									
Uses sleep medication	OR	0.95 (0.75, 1.19)	0.638	0.88 (0.70, 1.10)	0.260	**0.62 (0.48, 0.80)**	**<0.001**	**0.52 (0.39, 0.71)**	**<0.001**
How often does not feel rested	OR	0.96 (0.82, 1.12)	0.580	**0.82 (0.70, 0.96)**	**0.013**	0.97 (0.81, 1.17)	0.771	0.90 (0.73, 1.11)	0.327
**Subjective perceptions of health**									
Poor eyesight	OR	0.97 (0.84, 1.13)	0.734	**0.84 (0.73, 0.98)**	**0.022**	0.87 (0.74, 1.04)	0.127	0.87 (0.71, 1.06)	0.163
Poor hearing	OR	0.95 (0.82, 1.11)	0.529	**0.85 (0.74, 0.98)**	**0.026**	**0.81 (0.69, 0.96)**	**0.014**	0.84 (0.70, 1.01)	0.065
Perceived difficulty with balance	OR	0.86 (0.74, 1.01)	0.067	**0.69 (0.60, 0.80)**	**<0.001**	**0.80 (0.67, 0.95)**	**0.012**	**0.78 (0.64, 0.95)**	**0.015**

Note. Reference category no engagement. All models adjusted for age, gender, race/ethnicity, marital status, education, employment, pension status, household income, assets, household size, neighborhood safety, neighborhood physical disorder, neighborhood social cohesion, and the baseline measure of the outcome. Bold text indicates $p<0.05$. Results weighted and based on 20 imputed datasets. IRR: incidence rate ratio from negative binomial regression model. OR: odds ratio from ordered logistic regression model (binary logistic regression for falls and sleep medication). β: standardized coefficient from linear regression model. All tests were two-sided. No adjustments were made for multiple comparisons.

Table S11B. Adjusted regression models testing longitudinal associations between different levels of leisure engagement and experiences of aging eight years later (part B).

		**Cognitive activities**				**Community activities**			
		**Monthly**		**Weekly**		**Monthly**		**Weekly**	
	Coef	Coef (95% CI)	p	Coef (95% CI)	p	Coef (95% CI)	p	Coef (95% CI)	p
**Daily functioning**									
Difficulties with ADLs	IRR	1.01 (0.75, 1.38)	0.932	0.93 (0.67, 1.30)	0.680	0.92 (0.79, 1.06)	0.234	1.19 (0.65, 2.16)	0.574
Difficulties with IADLs	IRR	1.01 (0.77, 1.34)	0.919	0.97 (0.71, 1.31)	0.825	0.95 (0.81, 1.11)	0.492	1.00 (0.54, 1.84)	0.998
Difficulties with mobility	OR	1.13 (0.87, 1.47)	0.349	1.11 (0.85, 1.46)	0.442	1.01 (0.91, 1.12)	0.902	0.76 (0.53, 1.11)	0.154
**Physical fitness**									
Strength	IRR	1.01 (0.95, 1.08)	0.673	1.01 (0.95, 1.08)	0.671	1.01 (0.99, 1.04)	0.240	**1.09 (1.01, 1.18)**	**0.025**
Gross motor function	IRR	1.00 (0.97, 1.03)	0.861	1.01 (0.97, 1.04)	0.754	**1.01 (1.00, 1.02)**	**0.008**	1.02 (0.98, 1.05)	0.409
Fine motor function	IRR	1.02 (0.99, 1.05)	0.133	1.02 (0.99, 1.05)	0.140	1.00 (0.99, 1.01)	0.904	0.98 (0.94, 1.02)	0.288
Falls	OR	1.09 (0.69, 1.73)	0.698	1.02 (0.63, 1.65)	0.927	1.01 (0.85, 1.19)	0.910	1.00 (0.54, 1.86)	0.998
Gait speed	β	0.05 (-0.15, 0.25)	0.654	0.07 (-0.13, 0.28)	0.489	0.02 (-0.05, 0.08)	0.595	-0.05 (-0.33, 0.23)	0.732
Lung function	β	0.06 (-0.03, 0.15)	0.158	0.05 (-0.04, 0.14)	0.298	0.03 (-0.01, 0.06)	0.134	-0.04 (-0.16, 0.08)	0.475
Grip strength	β	0.05 (-0.03, 0.12)	0.251	0.03 (-0.05, 0.11)	0.483	-0.02 (-0.05, 0.01)	0.172	-0.09 (-0.21, 0.04)	0.173
Static balance	OR	1.23 (0.91, 1.66)	0.178	1.11 (0.80, 1.53)	0.534	1.07 (0.94, 1.22)	0.287	0.89 (0.47, 1.67)	0.720
**Long-term physical health problems**									
Chronic health conditions	OR	0.98 (0.76, 1.26)	0.867	1.04 (0.79, 1.36)	0.783	1.06 (0.95, 1.18)	0.309	1.17 (0.81, 1.70)	0.400
Degree of persistent pain	OR	1.02 (0.79, 1.33)	0.854	0.96 (0.73, 1.26)	0.765	**0.84 (0.75, 0.95)**	**0.004**	1.08 (0.67, 1.74)	0.758
**Heart health**									
Systolic blood pressure	OR	0.98 (0.75, 1.28)	0.889	1.01 (0.76, 1.34)	0.928	0.98 (0.87, 1.10)	0.731	1.58 (0.96, 2.59)	0.070
Diastolic blood pressure	OR	0.93 (0.69, 1.26)	0.648	1.03 (0.75, 1.42)	0.865	0.90 (0.78, 1.03)	0.131	1.35 (0.74, 2.47)	0.330
Pulse	β	-0.06 (-0.20, 0.08)	0.395	-0.03 (-0.17, 0.11)	0.666	-0.03 (-0.08, 0.03)	0.335	0.05 (-0.20, 0.30)	0.690
**Weight**									
BMI	OR	1.23 (0.86, 1.76)	0.246	1.31 (0.90, 1.90)	0.160	1.01 (0.87, 1.16)	0.912	1.07 (0.60, 1.92)	0.809
Waist circumference	β	0.01 (-0.06, 0.09)	0.771	0.02 (-0.06, 0.10)	0.681	0.00 (-0.03, 0.03)	0.999	0.02 (-0.12, 0.16)	0.796
**Sleep**									
Uses sleep medication	OR	0.88 (0.63, 1.23)	0.452	0.80 (0.55, 1.15)	0.228	1.11 (0.93, 1.33)	0.232	1.22 (0.67, 2.22)	0.521
How often does not feel rested	OR	1.03 (0.80, 1.33)	0.803	0.86 (0.66, 1.12)	0.258	0.92 (0.82, 1.03)	0.154	1.00 (0.63, 1.61)	0.986
**Subjective perceptions of health**									
Poor eyesight	OR	0.78 (0.63, 0.98)	0.034	**0.73 (0.58, 0.93)**	**0.011**	0.91 (0.82, 1.00)	0.061	0.85 (0.55, 1.31)	0.452
Poor hearing	OR	1.00 (0.80, 1.26)	0.974	0.94 (0.74, 1.20)	0.630	0.95 (0.85, 1.05)	0.283	0.94 (0.60, 1.45)	0.768
Perceived difficulty with balance	OR	1.12 (0.88, 1.42)	0.351	1.15 (0.90, 1.49)	0.266	0.98 (0.88, 1.08)	0.660	0.94 (0.61, 1.46)	0.796

Note. Reference category no engagement. All models adjusted for age, gender, race/ethnicity, marital status, education, employment, pension status, household income, assets, household size, neighborhood safety, neighborhood physical disorder, neighborhood social cohesion, and the baseline measure of the outcome. Bold text indicates $p<0.05$. Results weighted and based on 20 imputed datasets. IRR: incidence rate ratio from negative binomial regression model. OR: odds ratio from ordered logistic regression model (binary logistic regression for falls and sleep medication). β: standardized coefficient from linear regression model. All tests were two-sided. No adjustments were made for multiple comparisons.

Table S12. Adjusted regression models testing longitudinal associations between leisure engagement and experiences of aging eight years later, after excluding participants with major chronic conditions at baseline.

			**Physical activities**		**Creative activities**		**Cognitive activities**		**Community activities**	
	N	Coef	Coef (95% CI)	p	Coef (95% CI)	p	Coef (95% CI)	p	Coef (95% CI)	p
**Daily functioning**										
Difficulties with ADLs	1460	IRR	0.94 (0.78, 1.13)	0.530	**0.77 (0.60, 0.99)**	**0.038**	1.04 (0.83, 1.32)	0.717	1.22 (0.94, 1.58)	0.130
Difficulties with IADLs	1460	IRR	1.13 (0.95, 1.35)	0.181	0.94 (0.69, 1.28)	0.688	1.02 (0.76, 1.37)	0.900	0.98 (0.75, 1.29)	0.912
Difficulties with mobility	1460	OR	**0.88 (0.82, 0.95)**	**0.002**	0.94 (0.84, 1.06)	0.308	1.05 (0.93, 1.18)	0.444	1.07 (0.93, 1.23)	0.373
**Physical fitness**										
Strength	1460	IRR	1.01 (0.99, 1.02)	0.315	**1.02 (1.00, 1.03)**	**0.028**	1.00 (0.98, 1.01)	0.548	1.01 (0.99, 1.03)	0.483
Gross motor function	1460	IRR	1.00 (1.00, 1.01)	0.298	1.01 (1.00, 1.01)	0.110	1.00 (0.99, 1.01)	0.704	1.00 (0.99, 1.00)	0.355
Fine motor function	1460	IRR	1.00 (1.00, 1.01)	0.417	**1.01 (1.00, 1.01)**	**0.029**	1.00 (0.99, 1.00)	0.248	0.99 (0.99, 1.00)	0.165
Falls	438	OR	0.97 (0.81, 1.17)	0.749	0.98 (0.73, 1.32)	0.907	0.96 (0.71, 1.29)	0.776	1.06 (0.75, 1.51)	0.732
Gait speed	395	β	0.00 (-0.04, 0.04)	0.951	0.08 (0.02, 0.15)	0.015	0.05 (-0.02, 0.12)	0.188	-0.06 (-0.15, 0.03)	0.208
Lung function	1330	β	0.01 (-0.01, 0.03)	0.360	-0.01 (-0.04, 0.03)	0.753	0.02 (-0.02, 0.05)	0.362	0.02 (-0.03, 0.06)	0.510
Grip strength	1330	β	0.01 (-0.01, 0.03)	0.369	0.00 (-0.03, 0.03)	0.962	0.01 (-0.02, 0.04)	0.451	-0.01 (-0.05, 0.03)	0.578
Static balance		OR	1.09 (0.97, 1.22)	0.139	1.10 (0.92, 1.31)	0.304	0.95 (0.79, 1.13)	0.542	0.82 (0.65, 1.02)	0.073
**Long-term physical health problems**										
Chronic health conditions	1460	OR	**0.85 (0.79, 0.92)**	**<0.001**	1.02 (0.91, 1.15)	0.708	1.03 (0.92, 1.16)	0.592	1.08 (0.95, 1.23)	0.245
Degree of persistent pain	1460	OR	0.91 (0.82, 1.02)	0.095	0.97 (0.82, 1.14)	0.675	0.91 (0.76, 1.09)	0.299	1.04 (0.84, 1.28)	0.745
**Heart health**										
Systolic blood pressure	1330	OR	1.06 (0.98, 1.16)	0.161	0.99 (0.87, 1.14)	0.913	1.08 (0.94, 1.24)	0.262	1.03 (0.87, 1.22)	0.766
Diastolic blood pressure	1330	OR	1.09 (0.98, 1.20)	0.108	0.95 (0.81, 1.11)	0.518	1.04 (0.90, 1.21)	0.559	0.86 (0.72, 1.01)	0.073
Pulse	1330	β	0.01 (-0.03, 0.04)	0.790	-0.01 (-0.07, 0.05)	0.770	-0.04 (-0.10, 0.02)	0.181	-0.02 (-0.10, 0.06)	0.631
**Weight**										
BMI	1330	OR	0.96 (0.86, 1.07)	0.443	1.06 (0.90, 1.25)	0.477	0.95 (0.80, 1.13)	0.555	1.13 (0.90, 1.40)	0.289
Waist circumference	1330	β	-0.01 (-0.03, 0.01)	0.472	0.02 (-0.02, 0.05)	0.288	0.00 (-0.03, 0.03)	0.909	0.02 (-0.02, 0.06)	0.248
**Sleep**										
Uses sleep medication	1460	OR	0.95 (0.81, 1.11)	0.500	**0.71 (0.54, 0.93)**	**0.014**	0.96 (0.76, 1.21)	0.740	1.04 (0.76, 1.42)	0.801
How often does not feel rested	1460	OR	1.01 (0.92, 1.11)	0.784	0.96 (0.83, 1.11)	0.608	0.91 (0.80, 1.05)	0.204	1.06 (0.90, 1.26)	0.479
**Subjective perceptions of health**										
Poor eyesight	1460	OR	0.93 (0.86, 1.00)	0.064	1.02 (0.90, 1.15)	0.805	0.90 (0.80, 1.01)	0.073	1.04 (0.92, 1.18)	0.497
Poor hearing	1460	OR	**0.93 (0.87, 1.00)**	**0.048**	1.04 (0.93, 1.17)	0.493	0.97 (0.87, 1.09)	0.631	0.91 (0.79, 1.05)	0.189
Perceived difficulty with balance	1460	OR	0.96 (0.89, 1.03)	0.255	0.94 (0.84, 1.05)	0.285	0.98 (0.87, 1.11)	0.738	1.04 (0.90, 1.21)	0.575

Note. All models adjusted for age, gender, race/ethnicity, marital status, education, employment, pension status, household income, assets, household size, neighborhood safety, neighborhood physical disorder, neighborhood social cohesion, and the baseline measure of the outcome. Bold text indicates $p<0.05$. Results weighted and based on 20 imputed datasets. IRR: incidence rate ratio from negative binomial regression model. OR: odds ratio from ordered logistic regression model (binary logistic regression for falls and sleep medication). β: standardized coefficient from linear regression model. All tests were two-sided. No adjustments were made for multiple comparisons.

Figure S1. Concurrent associations between frequency of engagement in the four domains of leisure engagement and experiences of aging.

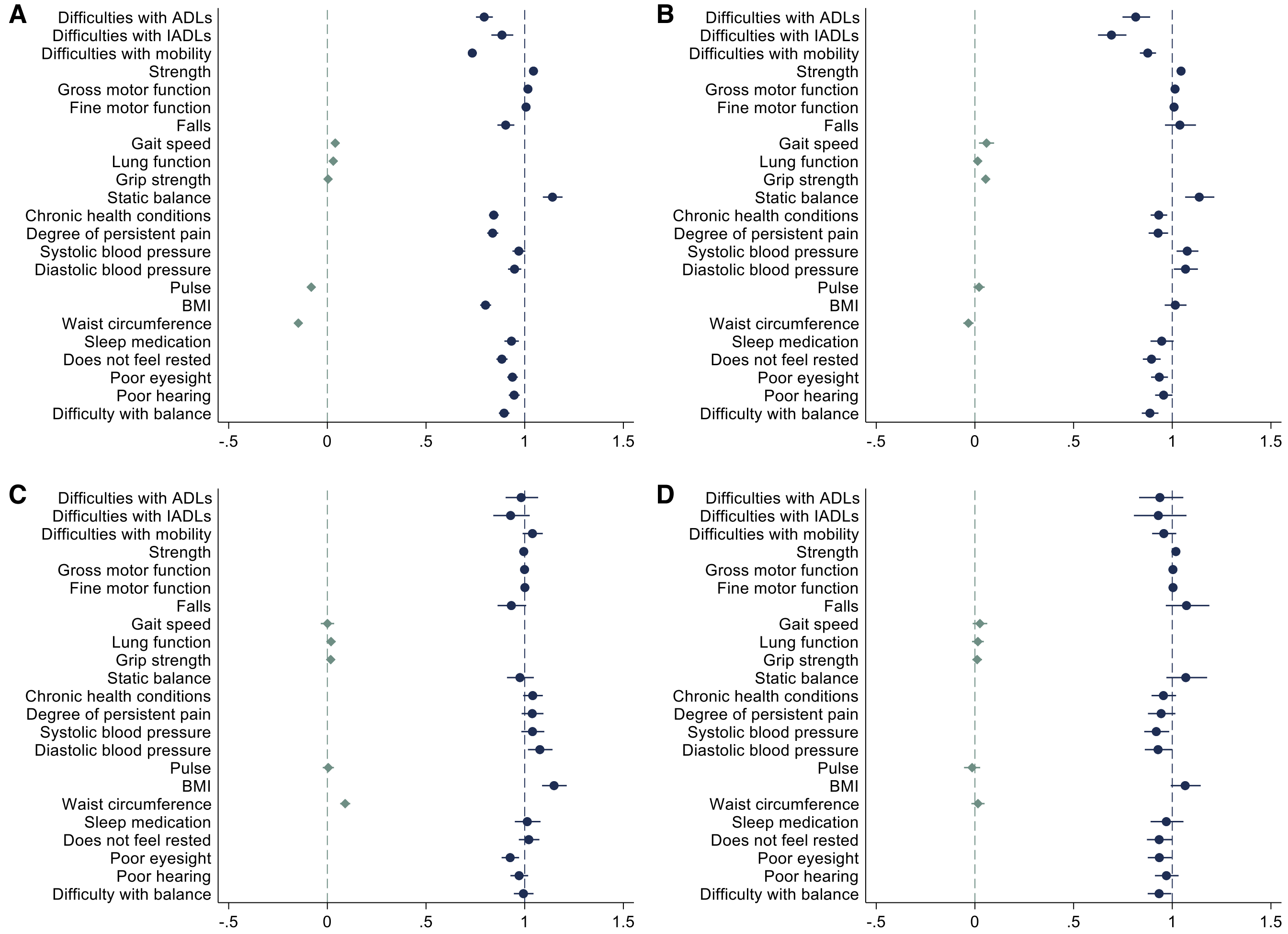

Note. Adjusted beta coefficients (diamonds), odds ratios (circles), incidence rate ratios (circles) from regression models testing the concurrent associations between frequency of engagement in the four domains of leisure engagement and experiences of aging. Data are presented as coefficients and accompanying 95% confidence intervals. Results adjusted for demographic, socioeconomic, and neighborhood covariates, weighted, and based on 20 imputed datasets. A) Physical activities. B) Creative activities. C) Cognitive activities. D) Community activities. Source data are provided as a Source Data file. Please see Table S1 for sample sizes, which differed across outcomes. P-values are presented in Table S8.

References

1. VanderWeele, T. J., Mathur, M. B. & Chen, Y. Outcome-Wide Longitudinal Designs for Causal Inference: A New Template for Empirical Studies. *Statistical Science* **35**, 437–466 (2020).
2. Vanderweele, T. J. Outcome-wide epidemiology. *Epidemiology* **28**, 399–402 (2017).
