# Supplementary material for: Leisure engagement in older age is related to objective and subjective experiences of aging
Source: Nat Commun. 2024 Feb 19;15:1499. doi: 10.1038/s41467-024-45877-w (PMC10876530; doi:10.1038/s41467-024-45877-w)
Supplement: Supplementary file 2 — Reporting Summary [file 41467_2024_45877_MOESM2_ESM.pdf]

nature portfolio

Corresponding author(s): Jessica K Bone

Last updated by author(s): Dec 20, 2023

Reporting Summary

Nature Portfolio wishes to improve the reproducibility of the work that we publish. This form provides structure for consistency and transparency in reporting. For further information on Nature Portfolio policies, see our Editorial Policies and the Editorial Policy Checklist.

Statistics

For all statistical analyses, confirm that the following items are present in the figure legend, table legend, main text, or Methods section.

n/a	Confirmed	
☐	☒	The exact sample size (*n*) for each experimental group/condition, given as a discrete number and unit of measurement
☐	☒	A statement on whether measurements were taken from distinct samples or whether the same sample was measured repeatedly
☐	☒	The statistical test(s) used AND whether they are one- or two-sided<br>*Only common tests should be described solely by name; describe more complex techniques in the Methods section.*
☐	☒	A description of all covariates tested
☐	☒	A description of any assumptions or corrections, such as tests of normality and adjustment for multiple comparisons
☐	☒	A full description of the statistical parameters including central tendency (e.g. means) or other basic estimates (e.g. regression coefficient) AND variation (e.g. standard deviation) or associated estimates of uncertainty (e.g. confidence intervals)
☐	☒	For null hypothesis testing, the test statistic (e.g. *F*, *t*, *r*) with confidence intervals, effect sizes, degrees of freedom and *P* value noted<br>*Give P values as exact values whenever suitable.*
☒	☐	For Bayesian analysis, information on the choice of priors and Markov chain Monte Carlo settings
☐	☒	For hierarchical and complex designs, identification of the appropriate level for tests and full reporting of outcomes
☐	☒	Estimates of effect sizes (e.g. Cohen's *d*, Pearson's *r*), indicating how they were calculated

Our web collection on statistics for biologists contains articles on many of the points above.

Software and code

Policy information about availability of computer code

Data collection	This research analysed data from the US Health and Retirement Study (HRS). No data collection software was used in this study.
Data analysis	Stata v17 was used for data analysis. All code for analyses in this study is publicly available online: https://doi.org/10.17605/OSF.IO/8NBXD

For manuscripts utilizing custom algorithms or software that are central to the research but not yet described in published literature, software must be made available to editors and reviewers. We strongly encourage code deposition in a community repository (e.g. GitHub). See the Nature Portfolio guidelines for submitting code & software for further information.

Data

Policy information about availability of data

All manuscripts must include a data availability statement. This statement should provide the following information, where applicable:

- Accession codes, unique identifiers, or web links for publicly available datasets
- A description of any restrictions on data availability
- For clinical datasets or third party data, please ensure that the statement adheres to our policy

Raw data are available from HRS (https://hrsdata.isr.umich.edu/data-products/public-survey-data) and the RAND Center for the Study of Aging (https://hrsdata.isr.umich.edu/data-products/rand). We are not data owners and therefore cannot share data in a public repository. Derived data supporting the findings of this study are available from the corresponding author JKB on request.

Research involving human participants, their data, or biological material

Policy information about studies with human participants or human data. See also policy information about sex, gender (identity/presentation), and sexual orientation and race, ethnicity and racism.

Reporting on sex and gender	HRS recorded gender as male/female. We included gender measured in this way in all adjusted analyses.
Reporting on race, ethnicity, or other socially relevant groupings	We measured race/ethnicity as White [including Caucasian], Black [including African American], Other [including American Indian, Alaskan Native, Asian or Pacific Islander, Hispanic, Other]). We included race/ethnicity in this way in all adjusted analyses.
Population characteristics	HRS is intended to be representative of the non-insitutionalised US population over 50
Recruitment	HRS used complex sampling to create a fully representative sample of individuals over the age of 50 in the US (full details available online)
Ethics oversight	This study has approval from the University of Florida (IRB201901792) and University College London Research Ethics Committee (project 18839/001)

Note that full information on the approval of the study protocol must also be provided in the manuscript.

Field-specific reporting

Please select the one below that is the best fit for your research. If you are not sure, read the appropriate sections before making your selection.

☐ Life sciences ☒ Behavioural & social sciences ☐ Ecological, evolutionary & environmental sciences

For a reference copy of the document with all sections, see nature.com/documents/nr-reporting-summary-flat.pdf

Behavioural & social sciences study design

All studies must disclose on these points even when the disclosure is negative.

Study description	This was a quantitative longitudinal study. We used regression models to test the associations between leisure engagement and subsequent experiences of aging in a cohort of older adults from the US.
Research sample	Participants were drawn from the Health and Retirement Study (HRS), a nationally representative study of more than 37,000 individuals over the age of 50 in the US. We included 8,771 older adults (55% female) who participated in HRS between 2006 and 2018. Ages ranged from 50 to 94 (mean=63.18, standard deviation [SD]=8.45). Overall, 85% were of White race/ethnicity, 10% Black/African American, and 5% identified as Other race/ethnicities, 71% were married, and 48% were retired.
Sampling strategy	HRS used a complex random sampling strategy. In this study, sample size was selected as participants who completed the HRS psychosocial questionnaire in 2008 or 2010, with complete data on leisure engagement, and also participated in the HRS core survey at our follow-up eight years later (2016/2018). Three additional limitations reduced our sample size further for some outcomes: completion of enhanced physical assessments at follow-up, aged 65 and over at follow-up, and both restrictions combined. Our sample sizes are similar to, or larger than, previous studies that have found evidence for associations between leisure engagement, physical health, and healthy aging.
Data collection	HRS collected data through a combination of pen and paper at in person or telephone interviews (most measures), pen and paper postal questionnaires (psychosocial questionnaire), and nurse-recoded outcomes at nurse visits (physical measures). Only participants (or person reporting on behalf of the participant) and researchers/nurses were present. As this is a secondary data analysis, all researchers and nurses collecting data were blind to the study hypothesis.
Timing	Data used in this study were collected between 2/2008 and 6/2019
Data exclusions	Participants were eligible to complete the HRS psychosocial questionnaire in 2008 or 2010, which we have combined to form the baseline of our study. Of the 15,405 participants who participated at baseline, 10,215 also participated in the HRS core survey at our follow-up eight years later (2016/2018) and were thus eligible for inclusion in our study. Of these, 8,893 participants had complete data on leisure engagement, and 8,771 also participated in the previous wave (in which health behavior covariates were measured), forming our final analytical sample for outcomes measured in the core survey.
Non-participation	For the HRS psychosocial questionnaire response rates were 85% in 2008 and 74% in 2010. For the subsequent core surveys, response rates were 83% in 2016 and 74% in 2018. Attrition and non-response were accounted for by HRS sample weights.
Randomization	Allocation was not random as this was a cohort study. Analyses controlled for a range of key demographic, socioeconomic, and neighborhood covariates and the outcome at baseline, as well as health and health behavior covariates in sensitivity analyses.

Reporting for specific materials, systems and methods

We require information from authors about some types of materials, experimental systems and methods used in many studies. Here, indicate whether each material, system or method listed is relevant to your study. If you are not sure if a list item applies to your research, read the appropriate section before selecting a response.

Materials & experimental systems

n/a	Involved in the study
☒	☐ Antibodies
☒	☐ Eukaryotic cell lines
☒	☐ Palaeontology and archaeology
☒	☐ Animals and other organisms
☒	☐ Clinical data
☒	☐ Dual use research of concern
☒	☐ Plants

Methods

n/a	Involved in the study
☒	☐ ChIP-seq
☒	☐ Flow cytometry
☒	☐ MRI-based neuroimaging

Plants

Seed stocks	*Report on the source of all seed stocks or other plant material used. If applicable, state the seed stock centre and catalogue number. If plant specimens were collected from the field, describe the collection location, date and sampling procedures.*
Novel plant genotypes	*Describe the methods by which all novel plant genotypes were produced. This includes those generated by transgenic approaches, gene editing, chemical/radiation-based mutagenesis and hybridization. For transgenic lines, describe the transformation method, the number of independent lines analyzed and the generation upon which experiments were performed. For gene-edited lines, describe the editor used, the endogenous sequence targeted for editing, the targeting guide RNA sequence (if applicable) and how the editor was applied.*
Authentication	*Describe any authentication procedures for each seed stock used or novel genotype generated. Describe any experiments used to assess the effect of a mutation and, where applicable, how potential secondary effects (e.g. second site T-DNA insertions, mosiacism, off-target gene editing) were examined.*
